# The emerging roles of N^6^-methyladenosine in osteoarthritis

**DOI:** 10.3389/fnmol.2022.1040699

**Published:** 2022-11-10

**Authors:** Hui Liu, Yi-Li Zheng, Xue-Qiang Wang

**Affiliations:** ^1^Department of Sport Rehabilitation, Shanghai University of Sport, Shanghai, China; ^2^Department of Rehabilitation Medicine, Shanghai Shangti Orthopaedic Hospital, Shanghai, China

**Keywords:** N^6^-methyladenosine, osteoarthritis, inflammation, apoptosis, senescence, autophagy, LncRNAs

## Abstract

Finding new biomarkers and molecular targets to guide OA treatment remains a significant challenge. One of the most frequent forms of RNA methylation, N^6^-methyladenosine (m^6^A), can affect gene expression and RNA transcription, processing, translation, and metabolism. Osteoarthritis (OA) can cause disability and pain degenerative disease, reduce the quality of life of the elderly, and increase the social and economic burden. Changes in m^6^A levels are crucial in OA progress. In this review, the discussion will concentrate on the role that m^6^A plays in OA occurrence and progression. The m^6^A involved in the OA process mainly includes METTL3 and FTO. Current studies on m^6^A and OA primarily focus on four signaling pathways, namely, NF-κB, LNCRNAs, ATG7, and Bcl2. m^6^A participates in these signaling pathways and affects cellular inflammation, apoptosis, senescence, and autophagy, thus controlling the OA process. The modification of m^6^A affects so many signaling pathways. For the treatment of OA, it may represent a viable new therapeutic target.

## Introduction

Osteoarthritis (OA) is a chronic, degenerative joint disease that occurs mainly in older individuals. It is a primary cause of disability and social expense (Hunter and Bierma-Zeinstra, 2019). It predominantly affects joint cartilage, resulting in joint discomfort, edema, and stiffness (Jang et al., 2021). Around $81 billion in direct medical expenses are incurred annually in the US due to arthritis (Yelin et al., 2007). From 2013 to 2015, The US Centers for Disease Control and Prevention gathered statistics that show that the incidence of osteoarthritis in American adults was 21%, or about 54.4 million (Barbour et al., 2017), and it is expected that by 2040, the number of American adults with osteoarthritis will reach 78.4 million (Hootman et al., 2016). According to a new study, 23.9% of Chinese people suffer from OA (Li et al., 2020). With the aging population and rising obesity rates, the prevalence of OA is projected to increase progressively. No specific drugs exist for the treatment of OA. Physical therapy and non-steroidal anti-inflammatory medicines (NSAIDs) remain the primary options for treating clinical symptoms, and severe cases may require joint replacement.

Epigenetic studies focus on non-coding RNA regulation, histone modification, and DNA methylation modification. The selective transcriptional expression of genes is regulated by DNA methylation modification. Histone modification occurs after protein translation. The histones that make up nucleosomes can be modified by various compounds, such as phosphorylation, acetylation, and methylation. Non-coding RNA regulates post-transcriptional expression of genes (Skvortsova et al., 2018). RNA methylation, as one of the essential contents of epigenetic research, refers to the methylation modification that occurs in different positions of RNA molecules. The two most prevalent RNA post-transcriptional alterations in eukaryotes are 6-methyladenine (N^6^-methyladenine, m^6^A) and 5-methylcytosine (C^5^-methylcytidine, m^5^C) (Nombela et al., 2021). Specifically, m^6^A regulates methylation and demethylation, which are controlled by several enzymes, to control RNA splicing, output, translation, and degradation. It then affects a variety of physiological and pathological processes (Zhao et al., 2017).

Previous studies (Zhang et al., 2020; Li et al., 2021) have investigated the relationship between m^6^A and osteoarthritis, but their studies were limited to m^6^A mediating inflammatory factors and regulating OA through the NF-κB pathway. In this review, we summarized the role of m^6^A in the occurrence and development of OA to better comprehend the connection between the two from four signaling pathways, namely, NF-κB, LNCRNAs, ATG7, and Bcl2. The anticipation includes the possibility that m^6^A will someday be used as a therapeutic target for OA.

## m^6^A

Early research found that m^6^A mainly occurs in the (G/A) (m^6^A) C sequence (Wang et al., 2014). Recent research has revealed that m^6^A predominantly appears in the termination codon and 3’UTR (Dominissini et al., 2012) and is translated in a cap-independent way near the 5′UTR (Karthiya and Khandelia, 2020). Thus, we can infer that m^6^A can affect gene expression, which in turn can affect RNA transcription, processing, translation, and metabolism. We emphasize the significance of this modification’s biological impact on the regulation of gene expression regulation (Roundtree et al., 2017), normal organismal development (Frye et al., 2018), and diseases (Hsu et al., 2017).

Mammalian cells undergo a dynamic and reversible m^6^A alteration mechanism (Zhou et al., 2020). Writers, erasers, and readers, which are the three essential components, can individually add, delete, or read m^6^A sites (Fu et al., 2014). The m^6^A modification procedure, which might potentially include reader involvement, can be launched by writers. Erasers, on the other hand, can promote the demethylation procedure. It has been discovered that these regulators are involved in RNA metabolic activities, such as selective cleavage, translation, stabilization, and miRNA treatment.

### Writers

Methyltransferases, also known as writers, are a group of critical catalytic enzymes that can cause m^6^A methylation of bases on mRNA. The complex that connects m^6^A to mRNA is made up of METTL3, METTL14, METTL16, WTAP, VIRMA, ZC3H13, HAKAI, and RBM15/15B, among other subunits. The complex’s two primary components are METTL3 and METTL14, which control the majority of m^6^A modifications (Geula et al., 2015).

Methyltransferase-like protein 3 (METTL3) and METTL14 can be combined to form a METTL3/14 heterocomplex. Among these, METTL3 is a catalytically active subunit; METTL14 is necessary for substrate recognition and promotes the complex’s binding to RNA (Wang et al., 2016). The function of Wilms tumor 1-associating protein (WTAP) is to bind METTL3/14 and induce them to recruit and locate to the substrate, and WTAP is needed for METTL3/14 nuclear speckle localization and m^6^A modification (Ping et al., 2014). METTL16 can be used as a “writer” to catalyze the process of m^6^A modification in mRNA, especially nascent mRNA. In the cytoplasm, METTL16 promotes the assembly of 80s ribosomes by directly binding to eIF3a/b (eukaryotic initiation factor 3a/b) and rRNAs, thus improving protein translation efficiency and promoting tumor genesis and growth (Su et al., 2022). According to one study, the 3’UTR and stop codon regions of mRNA are preferentially methylated by the Vir like m^6^A methyltransferase associated (VIRMA) enzyme (Yue et al., 2018). The complex is promoted to become nuclear localized by the zinc finger CCCH-type containing 13 (ZC3H13), which also induces the transfer of the complex into the nucleus (Wen et al., 2018). RNA binding motif protein 15/15B (RBM15/15B) combines with the enrichment region of uracil to promote the methylation of some RNAs (Patil et al., 2016).

### Erasers

Demethylases are methyl group-removing enzymes that are also referred to as erasers. Fat mass and obesity-associated protein (FTO) or α-ketoglutarate-dependent dioxygenase alkB homolog 5 (ALKBH5) can reverse m^6^A methylation, and the two proteins are known as m^6^A erasers. Jia et al. (2011) confirmed for the first time in the world that FTO protein is a very important demethylase in DNA and RNA. FTO and ALKBH5 promote the demethylation of m^6^A in a Fe (II)- and -ketoglutaric acid-dependent manner. They initially oxidized m^6^A to produce N^6^-hydroxymethyladenosine (hm^6^A), which is subsequently transformed into N^6^-formyladenosine (f^6^A). The demethylation procedure was finished when f^6^A changed into adenosine (A) (Wang et al., 2020).

The core domain of FTO protein is comparable with the ALKB protein family, but the unique long loop at C-terminal is different from other proteins of the ALKB family. The ability of FTO proteins to demethylate the methylated single-stranded DNA or RNA is due to this specific domain (Gerken et al., 2007). FTO is relevant to human ponderal growth, obesity, and disease (Zhou et al., 2017). Once the transcription level of the FTO gene becomes abnormal, it will cause a variety of conditions, such as acute myeloid leukemia (Wei et al., 2018). ALKBH5 is the second m^6^A demethylase to be discovered (Zheng et al., 2013). Almost all tissues express ALKBH5, and it is essential for mouse spermatogenesis (Aik et al., 2014). However, ALKBH5 and FTO expression levels varied in various tissues. For instance, in mice, ALKHB5 is predominately expressed in the testes and female ovaries (Zheng et al., 2013). In contrast, FTO is predominately expressed in the brain, which may be related to various biochemical pathways in which they are involved. The target RNA is moved from the nucleus to the cytoplasm when ALKBH5 is knocked out in human cell lines (Zheng et al., 2013). Nearly all published studies about ALKBH5 function have identified similar molecular pathways. ALKBH5 modulates some transcripts of 3’UTR m^6^A, mediates hypoxia-inducible factor (HIF)-dependent breast CSC phenotype, and regulates glioblastoma growth and carcinogenesis *via* the ALKBH5-FOXM1 pathway, thereby indicating its essential role in tumorigenesis (Zhang et al., 2017).

### Readers

To perform specific biological functions, a reader, which is also referred to as a particular RNA binding protein-methylated reading protein, is necessary for m^6^A-modified mRNA. By using an RNA pull-down assay, several reading proteins were discovered. These consist of YTH domain-containing proteins, nuclear heterogeneous ribosomal proteins (hnRNP), insulin-like growth factor 2 mRNA-binding proteins (IGF2BPs), and eukaryotic initiation factor (eIF) (He et al., 2019). These reading proteins have several roles, such as binding selectively to the m^6^A methylation region, diminishing homologous binding to RNA-binding proteins, and changing the secondary structure of RNA to affect protein-RNA interactions. A couple of proteins with YTH domains are the YTH domain-containing protein 1–2 (YTHDC1-2) and YTH N^6^-methyladenosine RNA binding protein 1–3 (YTHDF1-3). The cytoplasm is where YTHDF1-3 primarily identifies m^6^A-modified mRNA, whereas the nucleus is where YTHDC1-2 mostly functions. YTHDF1 promotes mRNA translation and protein synthesis by affecting the translation mechanism (Wang et al., 2015). By reducing its stability, YTHDF2 can regulate the rate of degradation of its target m^6^A transcript (Li et al., 2018). As a cofactor, target transcripts are translated and degraded more quickly in cells when YTHDF3 interacts with YTHDF1 and YTHDF2 (Shi et al., 2017). Proteins that contain YTH domains include YTHDC1-2. YTHDC1, which is located in the nucleus, has several functions, such as attracting some splicing factors to modulate mRNA splicing (Kasowitz et al., 2018), boosting mRNA output (Roundtree et al., 2017), and accelerating the decay of some transcripts (Shima et al., 2017). YTHDC2 modulates mRNA stability, translation (Mao et al., 2019), and spermatogenesis (Hsu et al., 2017). HnRNPC, hnRNPG, and hnRNPA2B1 are hnRNPs members, which can control how target transcripts are processed and spliced and also bind to some structures of RNA, which are mediated by m^6^A. The m^6^A reconstructs the local RNA structure and regulates the interaction between RNA- proteins. This phenomenon is called the m^6^A switch (Liu et al., 2015). IGF2BPs enhance RNA expression by promoting the stability of RNA (Muller et al., 2019).

## Osteoarthritis pathogenesis

Previously, OA was considered to be essentially a disease of mechanical damage, in which chronic loading and impaired joint biomechanics lead to the destruction of joint cartilage and inflammation, followed by stiffness, swelling, and reduced mobility. Studies suggested that OA is a more complex process regulated by inflammation and metabolic factors. At the cellular and molecular levels, OA is characterized by a transition from a healthy homeostasis state to a catabolic state (Chen et al., 2017). Articular cartilage is made up of chondrocytes, fibers, and stroma. Chondrocyte, the only type of cell found in cartilage, is responsible for generating and maintaining the cartilage matrix, which is mainly composed of type II collagen (coll-II) and proteoglycans (Glyn-Jones et al., 2015). In addition, chondrocytes are encapsulated in a rich extracellular matrix (ECM) and lack vascular, nerve, and lymphatic tissue (He et al., 2020). Changes in these components affect the dynamic balance of cartilage. At the molecular level, one of the significant causes of cartilage degeneration is the imbalance in ECM synthesis and breakdown in articular chondrocytes. Inflammatory mediators contribute to the degradation of cartilage ECM. These pro-inflammatory cytokines’ aberrant expression results in altered chondrocyte phenotypes. Interleukin-1 (IL)-1β and tumor necrosis factor-alpha (TNF-α) are two examples of conventionally pro-inflammatory cytokines that are elevated in the OA animal model. By activating the nuclear factor-κB (NF-κB) signaling pathway, they stimulate cellular inflammatory responses; they lead to extracellular matrix degradation by upregulation of matrix metalloproteinases (MMPs) (Choi et al., 2019). With age, the immune and surveillance functions are significantly weakened, and the aging cells cannot be effectively phagocytosed or cleared in time, resulting in accumulation (He and Sharpless, 2017; Mas-Bargues et al., 2021). Some inflammatory factors, such as IL-6 and IL-8, can accelerate cell aging in the way of autocrine, leading to cell apoptosis, thus accelerating the progression of OA (Soto-Gamez and Demaria, 2017). LncRNAs are functionally implicated throughout the whole life cycle of chondrocytes and in relation to OA progression. Over 20 different lncRNAs regulate OA pathogenesis by controlling the ECM degradation, chondrocyte activity, immunological response, and angiogenesis (Sun et al., 2019). The upregulation of MMP-13 and ADAMTS5 in OA is correlated with the expression of some lncRNAs, such as H19 (H19 imprinted maternally expressed transcript), and CTD-2574D22.4 (Xing et al., 2014).

## The role of m^6^A in OA pathway

m^6^A modifications are linked to OA. However, its biochemical mechanism and functional features are ambiguous. Thus, we summarize the existing evidence regarding m^6^A’s pleiotropic role in OA (Table 1).

**Table 1:** Roles of m^6^A key regulator in osteoarthritis.

m^6^A regulator	Expression	Cell type	Sample	Pathway	Impact on OA progress	Biological function	References
METTL3	Down	ATDC5 cell	C57BL/6 male mice	METTL3/NF-κB	Delay	Decrease the level of inflammatory factors, inhibit cellular inflammatory response, inhibit ECM degradation, and inhibit apoptosis	Liu et al. (2019)
METTL3	Up	SW1353 cell	/	METTL3/NF-κB	Acceleration	Reduced inflammatory factor levels and promote ECM degradation	Sang et al. (2021)
FTO	Down	Articular chondrocytes	MIA-induced mouse model	FTO/AC008/mir-328-3p	Acceleration	Reduces the viability of chondrocytes and increases apoptosis and ECM degradation	Yang et al. (2021)
METTL3	Up	FLSs	DMM mouse model	METTL3/ATG7/GATA4	Acceleration	Promote cell senescence and promote the development of osteoarthritis	Chen et al. (2022)
METTL3	Up	ATDC5 cell	C57BL/6 male mice	METTL3/YTHDF1/Bcl2	Delay	Inhibition of chondrocyte apoptosis and autophagy	He et al. (2022)
METTL3	Up	Chondrocytes	/	METTL3/LINC00680/ SIRT1	Acceleration	Promote ECM degradation and the proliferation repression	Ren et al. (2022)

DMM, destabilization of the medial meniscus; ECM, extracellular matrix; FLSs, fibroblast like synoviocytes; FTO, fat mass and obesity-associated protein; METTL3, Methyltransferase-like protein 3; MIA, monosodium iodoacetate; NF-κB, nuclear factor-κ.

### m^6^A regulates OA by mediating inflammation

NF-κB has been recognized as a typical proinflammatory signaling pathway for more than 30 years based on its role in the expression of pro-inflammatory cytokines and other pro-inflammatory genes. This pathway regulates immune-related pathways and affects cell survival, differentiation, and proliferation (Hayden and Ghosh, 2008). Liu et al. (2019) indicated a functional way of METTL3 in the process and underlying mechanisms of OA. METTL3 is expressed differently in various cells and tissues under varied experimental conditions, but METTL3 affects the development of OA by modulating the NF-κB signaling and ECM synthesis in chondrocytes. To simulate the inflammatory state *in vitro*, the inflammatory response of ATDC5 cells was stimulated by interfering with IL-1β. According to the findings, METTL3 expression was elevated in a dose-dependent manner. Inflammatory cytokines IL and TNF-α were also measured in terms of mRNA levels. Interfering with METTL3 silencing considerably decreased the expression of these molecules. By silencing the expression of METTL3, phosphorylated-p65 (p-p65), and p-κBα, NF-κB was inactivated. Silencing METTL3 with shRNA reduced the inflammatory cytokine levels and inhibited NF-κB signaling in chondrocytes, which delayed OA development. Moreover, METTL3 silencing decreased the expression of MMP13, and increased the expression of Aggrecan, thereby inhibiting ECM degradation. However, Sang et al. (2021) showed different results when they treated SW1353 cells with IL-1β. They found a decrease in METTL3 expression. Besides increasing the expression of P-65 and phosphorylated extracellular signal-regulated kinase (p-ERK), overexpression of METTL3 can also activate the NF-B signaling pathway and lower the levels of inflammatory cytokines. At the levels of mRNA and protein expression, MMP13, tissue inhibitors of metalloprotease (TIMP)-1, and TIMP-2 were decreased by METTL3 overexpression, but elevated levels of MMP1 and MMP3 increased ECM degradation. The different experimental results may be due to the different cell models selected. ATDC5 and SW1353 cells cannot wholly simulate the primary articular chondrocytes. Moreover, because collecting articular cartilage from people without OA is unethical, Sang et al. (2021) collected articular cartilage from patients undergoing hip replacement surgery for femoral neck fracture as normal control, but whether it can reflect the expression of METTL3 in normal human should be reconsidered. Liu et al. (2019) did not verify the expression of clinical OA, which may not reflect the real expression of OA patients.

### m^6^A regulates OA by mediating lncRNAs

An increasing number of studies indicated that m^6^A and long non-coding RNA (lncRNA) play a role in OA disease. However, the relationship between m^6^A and lncRNA’s biological role and clinical implications remain unclear. Yang et al. (2021) identified differential expression between normal and osteoarthritic cartilage by microarray analysis. AC008, Aquaporin 1 (AQP1) and ANK human gene (ANKH) are highly expressed in human OA. The high levels of AC008 and AQP1/ANKH expression can reduce the vitality of articular cartilage and promote chondrocyte apoptosis. AC008 promoted the expression of MMP13 and a disintegrin and metalloproteinase with thrombospondin motifs (ADAMTS)-5 and inhibited the expression of collagen type II alpha 1 (COL2A1) and Aggrecan, thus promoting ECM degradation. Through bioinformatics analysis, RNA immunoprecipitation (RIP), and luciferase analysis, it was observed that mir-328-3p might be targeting AQP1/ANKH, and AC008 plays the role of ceRNA through the sponge mir-328-3p. Thus, AC008 indirectly inhibits the effect of mir-328-3p on AQP1/ANKH. In addition, high expression of FTO in primary chondrocytes can down-regulate AC008 transcription, leading to instability of AC008, thus delaying the progression of OA. However, the low expression of FTO in OA leads to the up-regulation of AC008 transcription, thus accelerating the progression of arthritis. Ren et al. (2022) confirmed that LINC00680 was increased in OA tissue and isolated primary chondrocytes stimulated by 1 l-1β. METTL3 binds to the m^6^A site of LINC00680 and upregulates its expression. In 1 l-1β-induced chondrocytes, LINC00680 knockdown facilitates cell proliferation and inhibits ECM degradation. In terms of mechanism, LINC00680 can inhibit the transcription and expression of COL2A1 while stimulating the transcription and expression of MMP13, thus aggravating the degradation process of ECM, and accelerating the development of arthritis. Moreover, SIRT1 and IGF2BP2 are enzymes related to chondrocyte apoptosis. When IGF2BP2 interacts with LINC00680, which is triggered by METTL3 at the m^6^A modification site, Sirt1 mRNA becomes more stable. A suggestion for the future treatment of OA is provided by the interplay between m^6^A and lncRNA.

### m^6^A regulates OA by mediating cell autophagy

Autophagy Related Protein 7 (ATG7) is an autophagy effector enzyme that regulates the cell cycle and apoptosis in senescent cells (Collier et al., 2021). Senescence of synovial and cartilage can be triggered by aged fibroblast-like synovial cells (FLSs), which can also cause cartilage dysfunction. To study how METTL3 affects the control of autophagy, Chen et al. (2022) collected synovial tissues from healthy individuals and OA patients. The outcomes demonstrated that m^6^A modification by METTL3 could weaken ATG7 RNA’s stability, reduce the expression of ATG7, induce the generation and elevation of senescence-associated secretory phenotype (SASP), promote the aging of the synovial tissue, and accelerate the progression of OA. METTL3 was silenced by siRNA on the destabilization of the medial meniscus (DMM) surgery mice. The autophagy flux of OA-FLS increased. SASP decreased. METTL3 silencing inhibited cell senescence. Moreover, GATA4, a new aging regulator, was confirmed to be significantly upregulated in FLSs both *in vitro* and *in vivo*. GATA4 gene knockout inhibited 1 l-1β secretion and significantly inhibited SASP expression.

### m^6^A regulates OA by mediating cell apoptosis

BCL2 is a pro-survival protein that participates in the regulation of apoptosis and plays an important role (Willimott and Wagner, 2010). He et al. (2022) proved that METTL3 decreased in both *in vivo* mouse models of temporomandibular joint (TMJ) OA and *in vitro* inflammation. They identified Bcl2 mRNA as a downstream target of METTL3 using m^6^A-RNA immunoprecipitation (MeRIP). By siRNA, targeting Bcl2 silenced it, and the level of pro-apoptotic factors, such as bax and cleaved-caspase3, increased. However, the levels of anti-apoptotic factors, such as P62 and SRY-Box Transcription Factor 9 (SOX9), decreased. The role of METTL3 overexpression in controlling chondrocyte apoptosis and autophagy was reversed by silencing Bcl2. When YTHDF1 was silenced, Bcl2 mRNA’s stability was drastically decreased, because Bcl2 mRNA was regulated by YTHDF1 in an m^6^A-dependent way. METTL3 enhances the stability of Bcl2 through YTHDF1, thereby providing new insight into the treatment of TMJ OA.

## Conclusion and future prospects

The m^6^A is so comprehensively distributed across tissues, compared with other tissues, the m^6^A methyl group in brain tissue is highly specific (Liu et al., 2020). The role of m^6^A in the central nervous system, hematopoietic system and reproductive system has been widely discussed, especially in cancer progression (Jiang et al., 2021). Because the effect of m^6^A is so widespread, it is uncertain whether the compound that will eventually be developed to bind to the m^6^A will affect m^6^A in other tissues of patients with OA and there is a lack of studies on the safety and efficacy of m^6^A.

**Figure 1:**
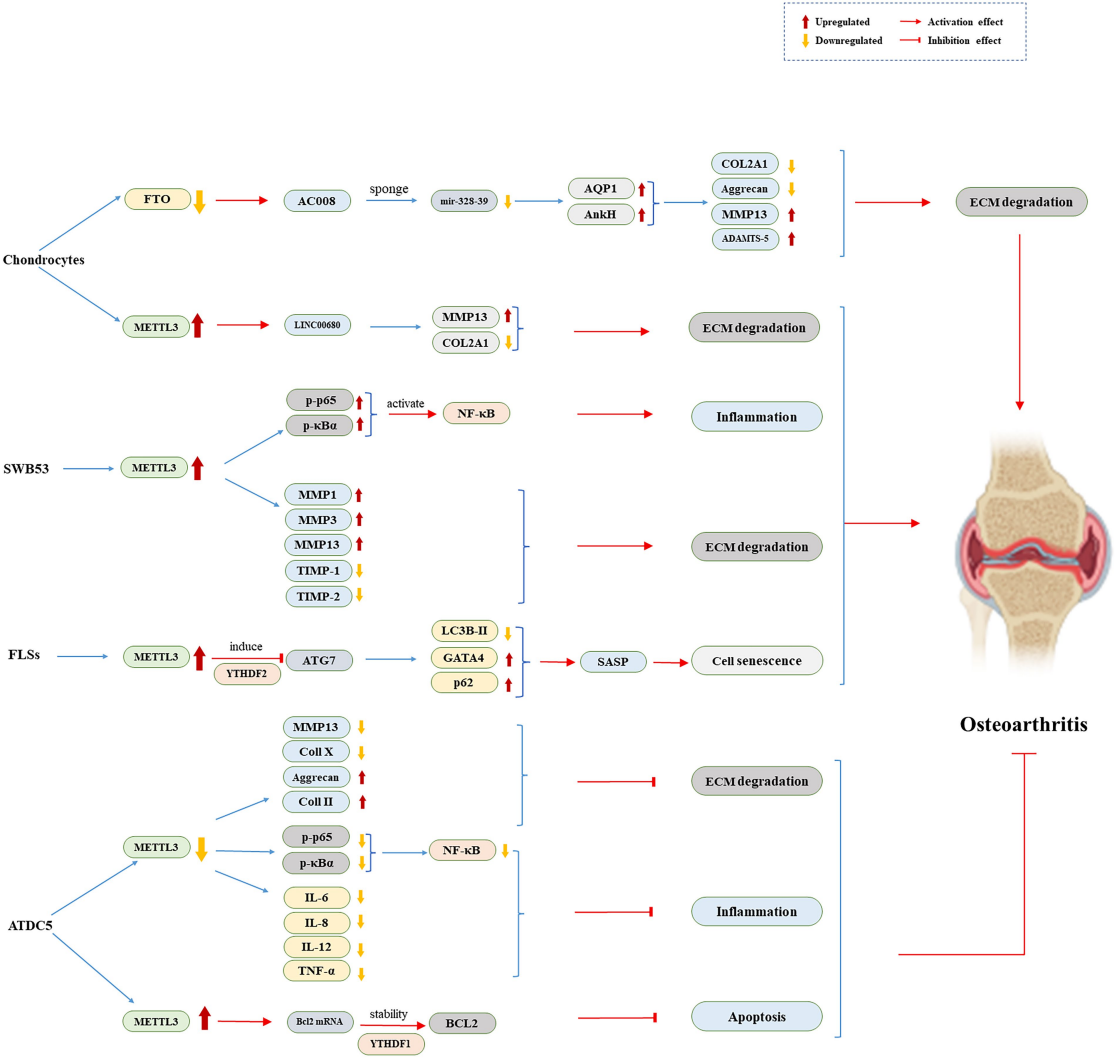
The role of m^6^A regulators in ATDC5, FLSs, SWB53, and Chondrocytes. ADAMTS-5, a disintegrin and metalloproteinase with thrombospondin motifs-5; ATG7, autophagy related protein 7; COL2A1, collagen type II alpha 1; Coll II, type II collagen; ECM, extracellular matrix; FLSs, fibroblast like synoviocytes; FTO, fat mass and obesity-associated protein; IL, interleukin; LC3B-II, microtubule-associated protein 1 light chain 3B-II; MMP, matrix metalloproteinase NF-κB, nuclear factor-κB; p-p65, phosphorylated-p65; TIMP, tissue inhibitors of metalloprotease; TNF-α, tumor necrosis factor alpha; YTHDF, YTH N^6^-methyladenosine RNA binding protein.

In this review, we emphasize the function of m^6^A and its regulatory mechanism in the genesis of OA (Figure 1). METTL3 plays a role in OA progression by mediating NF-κB signaling way and ECM synthesis in chondrocytes (Liu et al., 2019; Sang et al., 2021). METTL3 connects to the LINC00680 m^6^A site and promotes its expression. LINC00680 knockdown increases cell proliferation while inhibiting ECM breakdown (Ren et al., 2022). Furthermore, inhibiting METTL3 decreases cell senescence (Chen et al., 2022). The role of METTL3 overexpression in controlling chondrocyte apoptosis and autophagy was reversed when Bcl2 was silenced (He et al., 2022). High FTO expression in primary chondrocytes can inhibit AC008 transcription, causing AC008 instability and slowing the onset of OA (Yang et al., 2021). m^6^A regulates the inflammatory response, senescence, apoptosis, and autophagy by mediating these four pathways, delaying inevitable cartilage destruction, and thus plays a disease-modifying role in controlling the OA process.

In summary, for OA treatment, m^6^A offers a new approach. However, more studies are needed to comprehend the mechanisms and the correlation between m^6^A modification and OA, and on the safety and efficacy. Then, the m^6^A might represent a viable new treatment target for OA.

## Author contributions

X-QW contributed to conceptualization, project administration, writing—review and editing, and funding acquisition. Y-LZ contributed to conceptualization, writing—review, and editing. HL contributed to writing—original draft preparation. All authors contributed to the article and approved the submitted version.

## Funding

The authors disclosed receipt of financial support from the following for the research, authorship and/or publication of this article: the National Natural Science Foundation of China (81871844), Shanghai Key Lab of Human Performance (Shanghai University of Sport, fund number: 11DZ2261100), Shanghai Frontiers Science Research Base of Exercise and Metabolic Health, Talent Development Fund of Shanghai Municipal (fund number: 2021081), Shanghai Clinical Research Center for Rehabilitation Medicine (fund number: 21MC1930200), and the Scientific and Technological Research Program of the Shanghai Science and Technology Committee (fund number: 21S31902400).

## Conflict of interest

The authors declare that the research was conducted in the absence of any commercial or financial relationships that could be construed as a potential conflict of interest.

## Publisher’s note

All claims expressed in this article are solely those of the authors and do not necessarily represent those of their affiliated organizations, or those of the publisher, the editors and the reviewers. Any product that may be evaluated in this article, or claim that may be made by its manufacturer, is not guaranteed or endorsed by the publisher.
